# Alpha Band Resting-State EEG Connectivity Is Associated With Non-verbal Intelligence

**DOI:** 10.3389/fnhum.2020.00010

**Published:** 2020-02-04

**Authors:** Ilya Zakharov, Anna Tabueva, Timofey Adamovich, Yulia Kovas, Sergey Malykh

**Affiliations:** ^1^Developmental Behavioral Genetics Laboratory, Psychological Institute of the Russian Academy of Education, Moscow, Russia; ^2^Department of Psychology, Goldsmiths University of London, London, United Kingdom; ^3^International Centre for Research in Human Development, Tomsk State University, Tomsk, Russia

**Keywords:** EEG, resting state, connectivity, intelligence, neural efficiency, graph theory

## Abstract

The aim of the present study was to investigate whether EEG resting state connectivity correlates with intelligence. One-hundred and sixty five participants took part in the study. Six minutes of eyes closed EEG resting state was recorded for each participant. Graph theoretical connectivity metrics were calculated separately for two well-established synchronization measures [weighted Phase Lag Index (wPLI) and Imaginary Coherence (iMCOH)] and for sensor- and source EEG space. Non-verbal intelligence was measured with Raven’s Progressive Matrices. In line with the Neural Efficiency Hypothesis, path lengths characteristics of the brain networks (Average and Characteristic Path lengths, Diameter and Closeness Centrality) within alpha band range were significantly correlated with non-verbal intelligence for sensor space but no for source space. According to our results, variance in non-verbal intelligence measure can be mainly explained by the graph metrics built from the networks that include both weak and strong connections between the nodes.

## Introduction

Information processing in the brain is reflected in brain oscillations (Ward, 2003; Buzsáki and Draguhn, 2004; Clayton et al., 2015; Sadaghiani and Kleinschmidt, 2016). However, it is still not clear how neurobiological factors actually contribute to more effective cognitive performance. One approach to understanding the relationship between brain functioning and cognition is the neural efficiency hypothesis of intelligence (Haier et al., 1988, 1992). According to this hypothesis, brains of more intelligent individuals work more efficiently when engaged in cognitive task performance as compared to those of less intelligent ones. In a seminal studies (Haier et al., 1988, 1992), using the Positron Emission Tomography (PET) method, participants with higher scores on Raven’s progressive matrices were found to consume less glucose comparing to participants with lower scores. Later these results were extended to more types of brain activity measures (EEG, fMRI and so on) and different types of tasks (see Neubauer and Fink, 2009 for review).

The neural efficiency hypothesis predicts that the level of cognitive abilities would be correlated to brain activity during cognitive load. However, it is still unclear whether the brain activity at rest can be a good predictor of individual differences in intelligence. It has been proposed that the most informative way to investigate resting state activity is the network neuroscience approach (Deary et al., 2010). This is because intelligence is not localized in a single area in the brain but rather operates through a distributed network (Bullmore and Sporns, 2009). According to this approach, the nervous system is a network of anatomically and functionally interconnected areas that form distinct functional systems operating in a coherent manner. The structure and dynamics of these complex systems and connectivity patterns within them can be studied with network modeling tools that originate from mathematical graph theory. “Efficiency” in case of network approach is defined in terms of the cost of transmitting information within the network. In particular, it appears that brain networks are organized in a way that achieves the maximum possible cost-efficiency: a topological structure that maximizes complexity while minimizing transmission costs (Bassett et al., 2010; Denève and Machens, 2016).

Connectivity patterns of the brain resting state activity have been shown to be highly stable for an individual (Finn et al., 2015) and could be used for prediction of various personality traits (e.g., temperament or creativity; Markett et al., 2013; Beaty et al., 2018) or associated with psychopathological states (see van den Heuvel and Sporns, 2019 for review). The growing consensus in this area of research includes several features of the topology of the brain networks important for intelligence: (1) neuronal ensembles incerebral cortex are organized into complex networks due to frequency specific oscillatory coupling (da Silva, 1991; Barahona and Pecora, 2002; Buzsaki, 2006; Senzai et al., 2019); (2) the brain network graphs of functional oscillatory activity patterns share cost-efficient “small-world” properties [meaning that there is only small number of steps from one node to any other (Sporns, 2007; Stam and van Straaten, 2012)]; (3) characteristics of frequency-specific networks architecture are unique for a person and can be used as the identifying “fingerprints” of the network with almost 100% accuracy (Finn et al., 2015; Yeh et al., 2016); (4) communication through neuronal coherence within neuronal networks represent the neural substrate for individual differences in cognitive processes (Fries, 2015).

However, data on the relationship between the brain resting state activity and individual level of intelligence is inconsistent. In some studies the brain resting state functional connectivity characteristics correlated to intelligence (Langer et al., 2012). However, a recent large-scale study of 1200 individuals from the Human Connectome Project failed to find any significant associations between measures of the brain resting state dynamics and several widely used intelligence measures (Kruschwitz et al., 2018). This lack of significant associations could be due to the method of assessment of functional connectivity. The study used fMRI BOLD signal oscillations which have poor temporal resolution (2–3 s, Logothetis, 2008). A number of studies have shown that brain oscillations of much higher frequency can play a significant role in cognition (Palva and Palva, 2011; Fries, 2015; Sockeel et al., 2016).

The aim of the present study was to replicate the association between graph metrics of EEG resting state brain connectivity and non-verbal intelligence, found by Langer et al. (2012); and to assess consistency of several widely used methods of calculating EEG connectivity.

## Materials and Methods

Graph connectivity metrics were assessed during resting state. In EEG, functional connectivity can be estimated for the oscillations directly recorded from electrodes (sensor space connectivity) or for the reconstructed sources of brain activity. In the present study we included graph connectivity metrics both for sensor and source EEG space. As signal estimates are spatially correlated, a leakage of electromagnetic activity into local source neighborhood often occurs. When the synchronization method ignores this effect, “false positive” findings typically arise. Various methods were proposed to overcome the spatial leakage problem (see Bastos and Schoffelen, 2016 for a review). In the present study we used two most popular measures designed to correct for spatial leakage to replicate our results: weighted Phase Lag Index (wPLI, Vinck et al., 2010) and Imaginary Coherence (iMCOH, Nolte et al., 2008).

**FIGURE 1:**
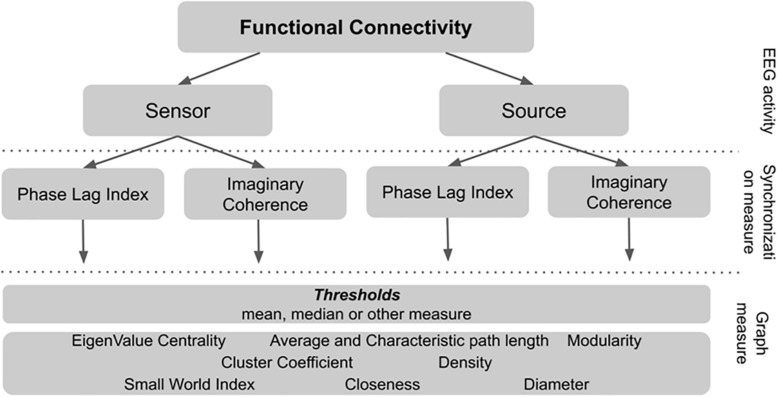
The plan of analysis of the connectivity metrics calculation.

To calculate graph connectivity metrics one has to choose the threshold synchronization value below which all the signal pairs are considered to be unrelated. In our study we systematically test several thresholds to understand how it affects the connectivity metrics and its relationship with non-verbal intelligence measure. The rationale for the network metrics choice and details of calculation are described in Supplementary Table S1. The plan of analysis is presented in Figure 1.

The non-verbal intelligence was measured with Raven’s Standard Progressive Matrices (Raven and Court, 1998).

### Participants

The participants were recruited via announcement in social networks (N = 165). They participated voluntarily without any monetary incentive. The exclusion criteria were any recorded history of psychiatric or neurological disorders and head trauma. Participants’ age ranged from 17 to 34 (M = 21.7, SD = 3.36, 30% identified as female). The majority of the participants were students or had a bachelor degree.

### Procedure

During resting state EEG acquisition all participants were instructed to sit still, think of nothing in particular and not to fall asleep for 10 min. Every 2 min the participants were asked to open or close their eyes with verbal instructions: “Now open your eyes,” “Now close your eyes.” Data with eyes closed were used for analysis in the present study.

The non-verbal intelligence was measured online before EEG recording with the shortened Raven’s matrices test (Raven and Court, 1998). The test consists of series of incomplete matrices. In each task participants should choose one of the eight suggested variants to complete the pattern. The original test, comprises six sets – A, B, C, D, E, and F. Within each set, the 12 items progressively become more difficult. We used four sets – C, D, E, and F. Sets C, D, and E contained six item each: 1st, 3rd, 5th, 7th, 9th, and 11th (items with even numbers were excluded); and set F contained 12 items. Thus, there were 30 items in total. Sets were presented in the following order: C – >D – >E – >F, where each set in turn became more difficult. A total sum of correct items was used as the measure of general cognitive ability.

### EEG Data Acquisition and Pre-processing

The EEG data was recorded from 64 active electrodes placed according to the international 10–10 system with Brain Products ActiChamp amplifier (BrainProducts, Munich, Germany). All experiments were conducted in a sound-attenuated and electrically shielded room with dim light. Impedance was kept under 25 kOhm with high conductive chloride gel. Approximate time for settling EEG was 15 min. The Brain Products PyCorder acquisition system was used for continuous recording without any filtering and continuously sampling at 500 Hz. The reference electrode was located at Cz. The data was re-referenced to the common reference after the recording and downsampled to 256 Hz. The data were filtered from 0.1 to 30 Hz and then re-referenced to an averaged reference and manually cleaned from artifacts, with noisy channels excluded. No more than 15% of the data was removed during artifact correction procedures. To remove blink and vertical eye-movement artifacts, independent component analysis (ICA) was performed on the following electrodes: VEOG — Fp1, HEOG — FT9 and FT10. After ICA, we topographically interpolated the excluded channels and conducted semiautomatic artifact rejection. The data were bandpassed into theta (4–8 Hz), alpha (8–13 Hz), beta1 (13–20 Hz), and beta2 (20–30 Hz) frequency ranges.

### EEG Data Analysis

#### Synchronization Measures

To assess synchronization between pair of signals two metrics were used. Both metrics were calculated with MNE Python software (Gramfort et al., 2014).

Weighted Phase Lag Index (Vinck et al., 2010; Hardmeier et al., 2014) is an extension of the PLI, which quantifies the asymmetry of the relative phase distribution. PLI ignores amplitude and is robustto spurious increase in the coherence between signals due to common sources of brain activity.

*PLI* = |⟨*sig*[ΔΦ(*tk*)]⟩|, where ΔΦ(*tk*) – phase shift between two signals.

By weighing each phase difference according to the magnitude of the lag, phase differences around zero only marginally contribute to the calculation of the wPLI.

Imaginary Coherence (Nolte et al., 2008) – is another attempt to solve common source problem. The method is based on the assumption, that common source activity is reflected in different channels simultaneously, without time-lag. iMOCH is designed so that it is sensitive to time-lagged processes only.

The iMCOH could be calculated as:

$$i\,c\,o\,h_{x\,y}\,\left(\omega \right)=\frac{I\,m\,\left(S_{x\,y}\,\left(\omega \right)\right)}{\sqrt{S_{x\,x}\,\left(\omega \right)\,S_{y\,y}\,\left(\omega \right)}},$$

$$\text{where}\,I\,m\,\left(S_{x\,y}\,\left(\omega \right)\right)-\mathrm{part}\,\mathrm{of}\,\mathrm{the}\,\mathrm{signal}\,\mathrm{with}\,\mathrm{time}\,\mathrm{shift}$$

#### Source Reconstruction

Source reconstruction was performed using standard source localization pipeline from MNE-package. First, source space with 503 sources for each hemisphere was created. Second, we used BEM (boundary-element model) to create three-layer model of the hemispheres. The three layers were inner skull, outer skull and outer skin. Conductivity of layers was standard for MNE package (0.3, 0.006, 0.3 for three layers accordingly). MNE exploit anatomical information from Free Surfer (Fuchs et al., 2001). Third, we constructed forward operator based on the source space and BEM model. Fourth, we created individual inverse operator for every participant with individual noise covariance matrix. Source reconstruction for each individual was performed with appropriate inverse operator using dSPM method (Dale et al., 2000).

### Connectivity Graph Measures

The connectivity metrics were chosen based on the reviews by Sporns and colleagues (Mišić and Sporns, 2016; Avena-Koenigsberger et al., 2018). In the present study we calculated the following graph connectivity metrics:

*“Small world index” (SWI)* – indexes the number of steps from one node to any other node within the network.
*Average and Characteristic Path Length* – the minimal number of edges that form a direct connection between two nodes (Average Path Length is based on the mean as the statistic, Characteristic Path Length – on the median).
*Cluster Coefficient* – a measure of the number of edges between a node’s nearest neighbors or the fraction of triangles around a node, and is a measure of functional segregation. High *C* represents clustered connectivity at the node.
*Modularity* – a measure of functional segregation, which quantifies how well the network can be subdivided into non-overlapping groups of nodes or modules.
*Diameter* – the greatest distance between any pair of nodes within the network.
*Eigenvector Centrality* – a measure of the influence of a node in a network. A high *eigenvector* score means that a node is connected to many nodes that themselves have high scores.
*Closeness Centrality* – a measure of *centrality* in a network, calculated as the reciprocal of the sum of the length of the shortest paths between the node and all other nodes in the *graph*.
Graph measures were calculated with igraph package^1^ for R (R Core Team, 2018).
The details of the calculation are presented in Supplementary Table S1.

## Statistical Approach

There is substantial variability in the possible routines of connectivity metrics calculation. There are several steps in EEG connectivity analysis where variability occurs. First, there is the alternative whether to use “raw” sensor-space EEG-signal or reconstruct and localize the source of EEG activity inside the brain. Second, there are different measures of synchronization between pairs of signals. Third, there is the convention to use only the pairs of signals with the strongest connections between them. However, the rationale to choose the “strong enough” threshold for synchronization measure is not explicitly and theoretically defined.

In our study we used two well-established but distinct measures of synchronization of the oscillatory brain activity (wPLI, Vinck et al., 2010; and iMCOH, Nolte et al., 2008). Synchronization was estimated for all pairs of EEG signals both for sensor and source EEG space separately for common EEG frequency bands (alpha, beta, and theta). In order to increase the number of investigated calculation alternatives and to decrease the number of multiple comparisons we developed the following approach used in the field of machine learning (Gareth, 2013). The whole sample was randomly divided into two subgroups: Test and Validation samples. Bootstrapped correlation coefficients were then separately calculated for the two samples– for non-verbal intelligence scores and all types of connectivity metrics. The median strength of connections within the person was used as the threshold (e.g., the 50% of the pairs with the highest synchronization estimates were used to calculate graph metrics). From this procedure we took only those metrics that were significantly correlated with non-verbal intelligence scores in both subsamples and both synchronization measures. To rule out possible impact of additional factors for these metrics, we also performed linear regression analysis with additional factors of sex and age of the participants. Another known variable, that can affect the EEG data is EEG spectral power. It was also added in the regression model.

The last step of the analysis was related to effect of thresholding on the connectivity metrics calculation. For the variables that remained significant after previous steps we calculated new metrics based on different synchronization thresholds (from 10 to 90% of the data preserved with the 10%-step). All the analyses were performed in R (R Core Team, 2018).

## Results

### EEG Sensor and Source Space Correlations

**FIGURE 2:**
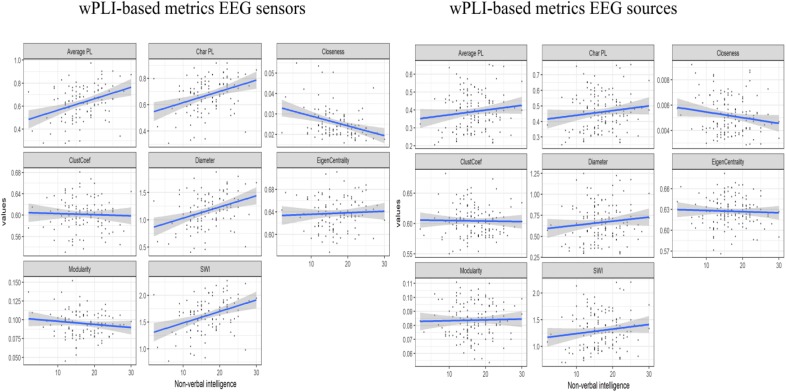
The scatterplots for the relationship between the wPLI-based connectivity metrics and non-verbal intelligence.

The consistent (repeated for different samples and synchronization measures) results were found only for alpha band EEG sensor space connectivity metrics. The bootstrapped correlations for the metrics in alpha band are presented in Table 1. The scatterplots for the relationship between wPLI-based metrics and intelligence are presented in Figure 2 (The descriptive statistics and correlations for other frequency bands, as well as scatterplots for iMCOH-based metrics can be seen in Supplementary Tables S2–S5 and Supplementary Figures S1, S2). There were a number of significant correlations between non-verbal intelligence and connectivity metrics for other frequency bands and EEG source space (see Supplementary Tables S2–S4), however, none of them were consistent for the different samples and synchronization measures.

**TABLE 1:** Bootstrapped correlations between alpha band connectivity metrics and non-verbal intelligence for test sample and validation sample.

	EEG sensor space				EEG source space			
Variable	WPLI		iMCOH		WPLI		iMCOH	
	Test	Validation	Test	Validation	Test	Validation	Test	Validation
Char PL	0.41**	0.31**	0.30**	0.32**	0.10	0.19	0.01	0.16
Average PL	0.43**	0.36**	0.30*	0.17	0.09	0.13	0.01	0.14
Clust coef.	–0.06	0.01	0.07	−0.28*	–0.00	–0.03	0.08	0.07
SWI	0.38**	0.27*	0.24	0.22	0.13	0.13	0.09	0.12
Modularity	–0.07	–0.20	–0.04	–0.09	–0.16	0.02	0.14	–0.18
Eigen. centrality	–0.06	0.16	–0.04	0.02	–0.02	–0.07	–0.05	0.01
Diameter	0.36**	0.29**	0.29*	0.29*	0.06	0.18	–0.00	0.16
Closeness	−0.35**	−0.28*	−0.38**	−0.31**	–0.17	–0.22	–0.09	–0.17

* indicates *p* < 0.05. ** indicates *p* < 0.01. Char PL, Characteristic Path Length; Average PL, Average Path Length; Clust Coef., Cluster coefficient; Eigen. Centrality, Eigenvector centrality.

At this step of analysis we were interested in the metrics that showed the same pattern of results both for wPLI and iMCOH measures and for both Test and Validation samples. Only four metrics calculated for alpha band EEG sensor space met these criteria (Average and Characteristic Path length, Diameter and Closeness). However, the collinearity analysis showed that multicollinaerity was present for these metrics (VIF > 5, Ringle et al., 2015). Accordingly, for the next step of the analysis we used only one of these metrics. We have chosen Characteristic Path Length due to its most straightforward theoretical interpretation.

The next step of the analysis was the linear regression analysis with sex, age and EEG spectral power as an additional variable. Both wPLI and iMCOH-based Characteristic Path Length remained statistically significant predictors of the level of non-verbal intelligence. The results are presented in Tables 2, 3.

**TABLE 2:** Regression results using non-verbal intelligence as the criterion and wPLI-based characteristic path length, sex, age, and EEG spectral power as predictors.

Predictor	*b*	*b* 95% CI [LL, UL]	*beta*	*beta* 95% CI [LL, UL]	*sr*^2^	*sr*^2^ 95% CI [LL, UL]	*r*	Fit
(Intercept)	8.77*	[0.69, 16.85]						
Characteristic PL	14.86**	[8.11, 21.61]	0.39	[0.21, 0.57]	0.13	[0.02, 0.24]	0.38**	
sex	–1.51	[−3.38, 0.36]	–0.14	[−0.31, 0.03]	0.02	[−0.02, 0.06]	–0.13	
Full years	0.02	[−0.20, 0.23]	0.01	[−0.16, 0.18]	0.00	[−0.00, 0.00]	–0.04	
alpha_power	–0.00	[−0.07, 0.06]	–0.01	[−0.18, 0.17]	0.00	[−0.00, 0.00]	0.09	
								*R^2^* = 0.168**
								95% CI[0.04, 0.26]

A significant *b*-weight indicates the beta-weight and semi-partial correlation are also significant. *b* represents unstandardized regression weights. *beta* indicates the standardized regression weights. *sr*^2^ represents the semi-partial correlation squared. *r* represents the zero-order correlation. LL and UL indicate the lower and upper limits of a confidence interval, respectively. * indicates *p* < 0.05. ** indicates *p* < 0.01.

**TABLE 3:** Regression results using non-verbal intelligence as the criterion and iMCOH based characteristic path length, sex, age, and EEG spectral power as predictors.

Predictor	*b*	*b* 95% CI [LL, UL]	*beta*	*beta* 95% CI [LL, UL]	*sr*^2^	*sr*^2^ 95% CI [LL, UL]	*r*	Fit
(Intercept)	14.80**	[8.15, 21.44]						
Characteristic PL	25.23**	[10.65, 39.81]	0.30	[0.13, 0.48]	0.08	[−0.01, 0.17]	0.32**	
sex	–1.16	[−3.08, 0.76]	–0.11	[−0.28, 0.07]	0.01	[−0.02, 0.04]	–0.12	
Full years	–0.01	[−0.21, 0.20]	–0.00	[−0.18, 0.17]	0.00	[−0.00, 0.00]	–0.02	
alpha_power	0.02	[−0.05, 0.08]	0.05	[−0.12, 0.23]	0.00	[−0.01, 0.02]	0.10	
								*R^2^* = 0.117**
								95% CI[0.01, 0.20]

A significant *b*-weight indicates the beta-weight and semi-partial correlation are also significant. *b* represents unstandardized regression weights. *beta* indicates that the standardized regression weights. *sr*^2^ represents the semi-partial correlation squared. *r* represents the zero-order correlation. LL and UL indicate the lower and upper limits of a confidence interval, respectively. ** indicates *p* < 0.01.

### Correlations for Different EEG Sensor-Space Connectivity Matrix Construction Thresholds

**FIGURE 3:**
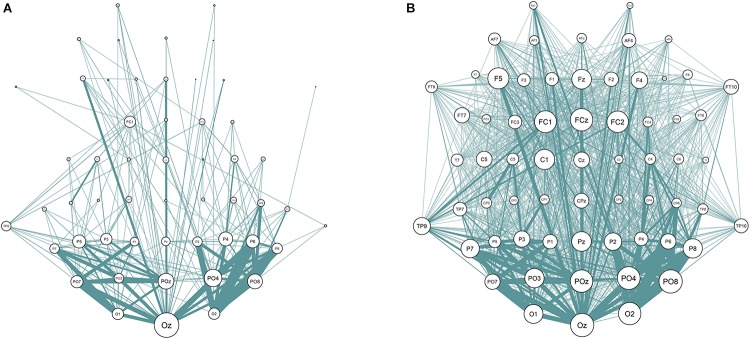
EEG Sensor space synchronization networks for wPLI. Connections strength lower than 90% **(A)** and 50% **(B)** of all connections removed. Node size represents node degree; edge width represents strength of the connection between two nodes.

One of the steps in the calculation of the connectivity metrics is thresholding procedure. Its main purpose is the increase in signal-to-noise ratio by deleting “weak” connections that do not contain any relevant physiological signal. The threshold values vary across studies considerably, which can lead to inconsistent results (Garrison et al., 2015). According to Sporns (2014) the average threshold is often chosen to delete the connections with the strength that lies at least below 75% of all connections. The discrepancies in the resulting networks with different percentiles (50th and 90th percentiles are taken as examples) can be seen in Figure 3.

**FIGURE 4:**
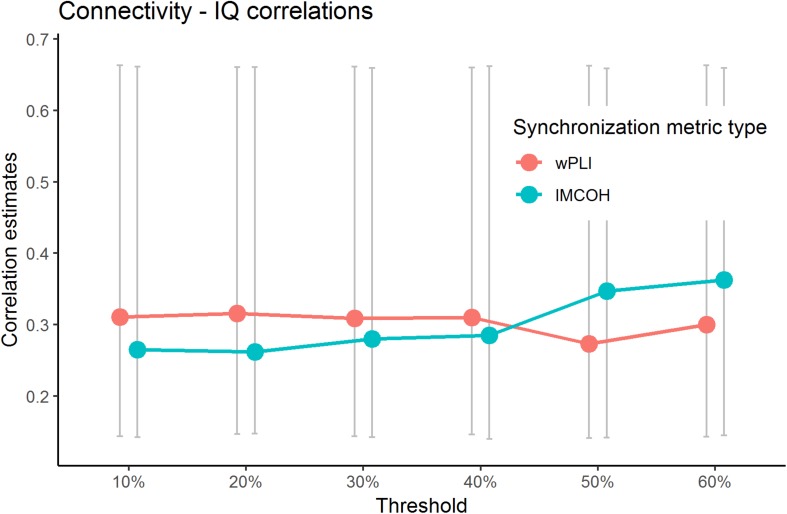
Significant correlations between wPLI and iMCOH connectivity metrics and non-verbal intelligence for different thresholds. Only correlations with metrics built with thresholds of 60% or lower are presented (*p* < 0.05, adjusted for multiple comparisons with FDR correction). Non-significant correlations (with 70% and higher threshold) for Characteristic PL are not presented. The bars represent 95% confidence intervals for bootstrapped correlations.

The current study addressed the problem of choosing the threshold explicitly. We have calculated the sensor space Characteristic Path length with ten different thresholds (from 10 to 90% with the 10%-step) for the test sample. The results are presented in Figure 3 (the detailed results are presented in Supplementary Table S6). No significant correlations with non-verbal intelligence were observed for the connectivity metrics calculated with 60% threshold or higher (i.e., with only strong connections between the nodes used to build the metric). The Characteristic Path Length metrics calculated with thresholds from 10 to 60% were significantly correlated with non-verbal intelligence, with *r* ranging from 0.24 to 0.36 (*p* < 0.05; adjusted for multiple comparisons with FDR correction). The pattern of these significant correlations is presented in Figure 4.

## Discussion

The neural efficiency hypothesis of intelligence is an important example of the neuroscience-based theories that promote understanding of psychological phenomena. One of the promising and well-suited methods for testing the neural efficiency hypothesis is the graph theoretical approach to the brain network analysis. However, in the recent large-scale fMRI study with 1096 participants, the connectivity-derived metrics of the brain dynamics were shown to be uncorrelated with various intelligence measures. fMRI is limited in capturing high frequency oscillations. A suitable measure of fast brain activity related to cognition is EEG due to its high temporal resolution. The current study tested the hypothesis that EEG-derived connectivity metrics is associated with non-verbal intelligence.

We found that average and characteristic path lengths, the diameter, and closeness centrality of the network in alpha band in EEG sensor space are correlated with the level of non-verbal intelligence in both test and validation samples. In the initial study by Langer et al. (2012) the negative correlation between intelligence and characteristic path length of the networks has been found. In contrast, in our study the correlation between non-verbal intelligence and graph metrics, related to path lengths characteristics, were positive (except closeness centrality, which is in inverse relationship with path lengths). The path length measures of a network we used are thought to be related to the integrative capacity of individual elements and the entire network (Avena-Koenigsberger et al., 2018). The average and characteristic (median) path lengths are related to the ease of the information transferring in the network. The diameter of the network measures the length of the shortest path between the most distanced nodes of a graph. The higher the diameter, the less linked a network tends to be. In general, the brain networks are supposed to be organized to ensure reliable and efficient communication, while minimizing the spent resources. Networks with longer paths are supposed to be energetically and metabolically more costly to be developed (Bullmore and Sporns, 2012). At the same time, in real architectures near-minimal pathway structure violates strict mathematical minimization criteria and require additional energetic cost (Rubinov et al., 2015; Betzel et al., 2016). This fact is compatible with the notion, that nervous systems were evolutionary selected not only for cost minimization, but also for topological integration (Sousa et al., 2017; Ardesch et al., 2019).

The importance of the integration of different brain areas can also explain another result of our study. We have found that the weak connections in the EEG sensor space yielded the most important information about the relationship between connectivity metrics and non-verbal intelligence. In connectivity studies mostly strong connections between the nodes have been accounted for, while weak connections were eliminated from calculation of the metrics. But, according to our data, variance in intelligence measurecan be mainly explained by the graph metrics built from the networks with not only strong, but weak to moderate strength of connections between the nodes as well. Weak EEG connections could represent additional abundant pathways that result in resilient communication within the network. Weak connections have been hypothesized to be important for stability of various types of networks (Granovetter, 1973; Pajevic and Plenz, 2012). Excessive number of pathways could be used to make the network less prone to bottlenecks and delays in the information transfer (Nassi and Callaway, 2009; Brandon et al., 2014. Several computational studies have demonstrated that geometrically embedded networks are, actually, characterized by physically short excessive connections that have an overall topology that increases the mean path length (Kaiser and Hilgetag, 2006). Research with macaque monkeys has shown that weak connections in the brainare important for neural cohesion (Goulas et al., 2015). Another possibility is that “a more intelligent brain” can engage larger amount of the distributed brain areas into the task solving process, which is in line with the parallel distribution processing theory (McClelland and Rumelhart, 1989; Bowers, 2017).

We have found that connectivity metrics of the brain networks oscillating within alpha frequency band range are sustainably correlated with non-verbal intelligence over wide variety of types of connectivity metrics, which is in line with the neural efficiency hypothesis. The EEG activity in the alpha range has been repeatedly associated with scores in various intelligence measures (Doppelmayr et al., 2002, 2005; Bazanova and Vernon, 2014). It has been hypothesized that the alpha band power reflects inhibition of non-essential processing (Klimesch et al., 2007). Thus, individual differences in the characteristics of resting-state alpha connectivity can indicate one’s ability to inhibit activity irrelevant to the task at hand, which can lead to higher task performance at intelligence tests. The brain oscillations are supposed to be the mechanism that synchronize the activity between different areas. The frequency of oscillations has been shown to be related to the spatial scale of the synchronization. Rhythms with higher frequency synchronize communication on relatively small spatial scales within the brain, while slower rhythms synchronize activity between more distant brain areas (Buzsaki, 2006; Fries, 2015). Therefore, the large-scale communication between the brain areas during resting state can be one of the key mechanisms underlying individual differences in cognitive functioning.

Our results are in line with recent diffusion tensor imaging study, where the preserving of weak connections on calculation of graph connectivity metrics was advocated for Civier et al. (2019), and with earlier fMRI study where thresholding has been shown to lead to inconsistent results (Garrison et al., 2015). The results suggest that common practice in research to eliminate weak connections may lead to missing important information. One way to avoid arbitrary thresholding is the data-driven approach to construction of the graphs, which can be based, for example, on the minimal spanning tree algorithms, (Dimitriadis et al., 2017, 2018). However, spanning-tree approach can be highly dependent on an initial state of the data and lead to different results with minor changes in the signal. Thus, the direct comparison of the results obtained with thresholding approaches and various data-driven approaches are needed.

Our study has a number of limitations. First, there is no consensus in the literature about the best measure of EEG synchronization, and the most stable source localization approach. In the current study we have chosen two of the most favorites synchronization measures and one paradigm of EEG source localization. Further research is needed to test whether our results can be replicated with other types of measures: (1) synchronization- orthogonalized envelope correlation; canonical coherence; bicoherence; phase shift invariant imaging of coherent sources (Ossadtchi et al., 2018,Vidaurre et al., 2019); and (2) localization – LORETA, FOCUSS, MUSIC (Jatoi et al., 2014).

Second, we hypothesize that the discrepancy in the association between intelligence level and EEG and fMRI-derived connectivity metrics can be attributed to higher EEG temporal resolution. In the present study we were not able to compare these methods directly. The combined EEG-fMRI study is needed to further investigate this question.

Lastly, in the initial studies, the neural efficiency hypothesis was based on the analysis of the brain activity during cognitive performance rather than resting state. To clarify whether the graph theoretical approach is a good measure of efficiency within neural networks under cognitive load, a replication of classical studies with the graph connectivity metrics calculation is needed.

Overall, we have found that the connectivity characteristics of the brain networks (particularly, oscillating within alpha range), derived from EEG resting state with graph theoretical approach, are significantly correlated with non-verbal intelligence. This is in line with the neural efficiency hypothesis. According to our results, weak EEG connections contain an important information about the brain activity. Therefore, it is possible that the widely used thresholding procedure can lead to increase in false negative results.

## Data Availability Statement

The datasets generated for this study are available on request to the corresponding author.

## Ethics Statement

This study received approval from the Ethics Committee of the Psychological Institute of the Russian Academy of Education. For all participants written informed consent was obtained.

## Author Contributions

IZ, AT, and SM contributed to the conception and design of the study. IZ and AT collected the data. IZ, AT, and TA performed the statistical analysis. IZ wrote the first draft of the manuscript. AT and TA assisted in writting the manuscript. YK and SM edited and finally approved the submitted manuscript. All authors read and approved of the submitted version of the manuscript.

## Conflict of Interest

The authors declare that the research was conducted in the absence of any commercial or financial relationships that could be construed as a potential conflict of interest.

## Footnotes

1 http://igraph.org
