# Supplementary material for: Alpha Band Resting-State EEG Connectivity Is Associated With Non-verbal Intelligence
Source: Front Hum Neurosci. 2020 Feb 4;14:10. doi: 10.3389/fnhum.2020.00010 (PMC7010914; doi:10.3389/fnhum.2020.00010)

Supplementary Material

**Supplementary Table 1.** The list of the network metrics

| **Path Length (Average and Characteristic)** | Path length is the minimum distance (number of edges) between two vertices. Those distances form a distribution, where **average** path length and **characteristic** path length are mean and median of this distribution  $l_{G}=\frac{1}{n\left( n-1 \right)}*\sum_{i\neq j} d\left( v_{i},v_{j} \right)$, where n – the number of vertices, and $d\left( v_{i},v_{j} \right)$ – shortest path from $v_{i}$ to $v_{j}.$ |
| --- | --- |
| **Closeness centrality** | Closeness centrality is another measure of the centrality, based on the idea than central nodes have shorter distances to other nodes. Closeness of the node is the inverse of the average path length from this node to all the others.  $C_{i}=\frac{1}{\sum d\left( i,j \right)}, where d\left( i,j \right)-distance from node i to node j$ |
| **Modularity** | Modularity is a measure of a division of a network into subgraphs (modules), which are characterized by more dense connections inside subgraphs than between them. For a defined number of clusters, modularity is:  $Q= \sum_{i=1}^{k} (e_{ii}-a_{i}^{2})$, где - $e_{ii}$probability edge is in module i, $a_{i}^{2}$ – probability of a random edge in the modulei  For undefined number of clusters:  $Q= \frac{1}{4m}\sum_{i,j} (A_{ij}-\frac{k_{i}k_{j}}{2m})$, where m – the number of vertices,$k_{i}andk_{J}-$degree of i and j nodes respectively, $A_{ij}$ – adjacency matrix. |
| **Cluster Coefficient** | Cluster coefficient is a measure of the tendency of graph nodes to create interconnected groups. For each node, cluster coefficient is a probability of two closest neighbours of this node being closest neighbours to each other:  $C_{avg}=\frac{1}{N}\sum_{i=1}^{N} \frac{2L_{i}}{k_{i}(k_{i}-1)}$, where $k_{i}$ – degree of node i, and $L_{i}$ – number of edges between neighbors of node i. |
| **Small World Index** | Small-world network is used to define the similarity of a graph to a small-world network. Is small-world network most nodes are not neighbours of one another, but could easily be reached via other neighbours via a small number of steps. SWI is defined through a comparison of graphs cluster coefficient and path length to random network with a similar degree  $\sigma= \frac{\frac{С}{С_{r}}}{\frac{L}{L_{r}}}$ |
| **Eigenvector centrality** | The concept of eigenvector centrality is based on an assumption about nodes of higher degree having greater influence on a network. Eigenvector centrality of high-degree node also depends on a number of connections with other high-degree nodes. Thus, higher eigenvector centrality means, that node is heavily connected with other high-degree nodes.  Eigenvector centrality is proportional to eigenvector centralities of neighbors and could be defined as:  $x_{i}=\frac{1}{⋋}*\sum_{x\in M(i)} x_{j}$ , where M(i) – neighbours of node x_i |
| **Diameter** | Diameter of a graph is the maximum possible shortest path length in a graph,  D = max$( d\left( v_{i},v_{j} \right))$ – where $d\left( v_{i},v_{j} \right)$shortest paths from $v_{i}$ to $v_{j}.$ |

**Supplementary Table 2.**

*Means and standard deviations for connectivity metrics*

|  | Alpha | | | | Beta1 | | Beta2 | | Theta | |
| --- | --- | --- | --- | --- | --- | --- | --- | --- | --- | --- |
| Variable | Test  Sample | | Validation  Sample | | Test  Sample | Validation  Sample | Test  Sample | Validation  Sample | Test  Sample | Validation  Sample |
|  | *M* | *SD* | *M* | *SD* | *M* | *SD* | *M* | *SD* | *M* | *SD* |
|  |  |  |  |  |  |  |  |  |  |  |
| 1.Non-verbal intelligence score | 17.45 | 4.80 | 17.31 | 5.25 |  |  |  |  |  |  |
| 2. Char PL | .15 | .06 | .14 | .05 | 0.06 | 0.02 | 0.04 | 0.02 | 0.07 | 0.03 |
| 3. Average PL | .13 | .05 | .12 | .04 | 0.05 | 0.02 | 0.04 | 0.02 | 0.07 | 0.03 |
| 4. ClustCoef | .55 | .04 | .55 | .04 | 0.56 | 0.03 | 0.55 | 0.03 | 0.55 | 0.03 |
| 5. SWI | 2.27 | 1.05 | 2.08 | .92 | 0.95 | 0.39 | 0.69 | 0.27 | 1.08 | 0.51 |
| 6. Modularity | .07 | .02 | .07 | .02 | 0.08 | 0.01 | 0.08 | 0.01 | 0.08 | 0.02 |
| 7. EigenCentrality | .65 | .03 | .65 | .04 | 0.65 | 0.03 | 0.66 | 0.04 | 0.67 | 0.03 |
| 8. Diameter | .24 | .11 | .24 | .10 | 0.10 | 0.04 | 0.07 | 0.03 | 0.12 | 0.06 |
| 9. Closeness | .02 | .009 | .02 | .01 | 0.28 | 0.11 | 0.38 | 0.13 | 0.24 | 0.08 |

*Note.* Char PL **-** Characteristic Path Length, Average Pl - Average Path Length, ClustCoef - Cluster coefficient, EigenCentrality - Eigenvector centrality.

**Supplementary Table 3.** Correlations beta1 band (13-20 Hz) connectivity metrics and non-verbal intelligence for Test Sample and Validation Sample

|  | | | | EEG Sensor space | | | | | EEG source space | | | |
| --- | --- | --- | --- | --- | --- | --- | --- | --- | --- | --- | --- | --- |
| Variable | | WPLI | | | iMCOH | | | WPLI | | | iMCOH | |
|  | Test | | Validation | Test | | Validation | Test | | Validation | Test | | Validation |
| **Char PL** | .14 | | .01 | .04 | | .16 | -.13 | | .04 | -.12 | | -.09 |
| **AveragePL** | .14 | | .00 | .04 | | .16 | -.14 | | -.06 | -.15 | | -.09 |
| **ClustCoef** | .25* | | .05 | -.07 | | -.05 | .16 | | .03 | .06 | | .05 |
| **Modularity** | -.029 | | -.08 | -.11 | | .13 | -.01 | | .06 | .06 | | -.31** |
| **EigenCentrality** | -.06 | | .13 | .11 | | .09 | .00 | | .16 | -.04 | | -.13 |
| **Diameter** | .19 | | .01 | .06 | | .14 | -.07 | | -.02 | .02 | | -.03 |
| **Closeness** | -.22 | | .02 | -.12 | | -.09 | .09 | | -.08 | .03 | | .05 |
| **SWI** | .08 | | .02 | .12 | | .011 | .04 | | -.05 | .02 | | -.08 |

*Note.* * indicates *p*< .05. ** indicates *p*< .01. Char PL **-** Characteristic Path Length, Average Pl - Average Path Length, ClustCoef - Cluster coefficient, EigenCentrality - Eigenvector centrality.

**Supplementary Table 4.** Correlations beta2 band (20-30 Hz) connectivity metrics and non-verbal intelligence for Test Sample and Validation Sample

|  | EEG Sensor space | | | | EEG source space | | | |
| --- | --- | --- | --- | --- | --- | --- | --- | --- |
| Variable | WPLI | | iMCOH | | WPLI | | iMCOH | |
|  | Test | Validation | Test | Validation | Test | Validation | Test | Validation |
| **Char PL** | -.22 | .02 | -.12 | -.09 | .09 | -.08 | .03 | .05 |
| **AveragePL** | .11 | -.04 | .23* | -.12 | .00 | -.07 | .01 | -.11 |
| **ClustCoef** | .17 | -.03 | .23* | -.10 | -.13 | -.02 | -.07 | -.05 |
| **Modularity** | .09 | .08 | -.02 | .09 | .02 | .35** | -.02 | .13 |
| **EigenCentrality** | -.10 | .11 | -.13 | .07 | .29** | .06 | .08 | -.07 |
| **Diameter** | -.09 | .06 | -.09 | .00 | -.19 | -.06 | .02 | -.15 |
| **Closeness** | .21 | .09 | .24* | -.10 | .01 | -.01 | .01 | -.09 |
| **SWI** | .11 | -.04 | .11 | -.10 | -.12 | -.07 | -.07 | -.02 |

*Note.* * indicates *p*< .05. ** indicates *p*< .01. Char PL **-** Characteristic Path Length, Average Pl - Average Path Length, ClustCoef - Cluster coefficient, EigenCentrality - Eigenvector centrality.

**Supplementary Table 5.** Correlations theta band (4-8 Hz) connectivity metrics and non-verbal intelligence for Test Sample and Validation Sample

|  | EEG Sensor space | | | | EEG source space | | | |
| --- | --- | --- | --- | --- | --- | --- | --- | --- |
| Variable | WPLI | | iMCOH | | WPLI | | iMCOH | |
|  | Test | Validation | Test | Validation | Test | Validation | Test | Validation |
| **Char PL** | .10 | .04 | .10 | -.17 | .11 | .07 | .09 | -.11 |
| **AveragePL** | .15 | -.02 | .07 | -.09 | .11 | .07 | .15 | .03 |
| **ClustCoef** | .12 | .15 | .16 | .15 | -.06 | .19 | -.27* | .10 |
| **Modularity** | .14 | -.03 | .11 | -.01 | .15 | .03 | .04 | .09 |
| **EigenCentrality** | .14 | -.04 | -.26* | -.03 | .22 | -.26* | .20 | -.16 |
| **Diameter** | .17 | .09 | .04 | -.09 | .05 | .05 | .07 | -.12 |
| **Closeness** | -.08 | .00 | -.06 | .09 | -.03 | .08 | -.00 | -.06 |
| **SWI** | .11 | -.07 | .02 | -.05 | -.03 | .08 | .03 | .06 |

*Note.* * indicates *p*< .05. ** indicates *p*< .01. Char PL **-** Characteristic Path Length, Average Pl - Average Path Length, ClustCoef - Cluster coefficient, EigenCentrality - Eigenvector centrality.

**Supplementary Table 6.** Correlations between wPLI and iMCOH connectivity metrics and non-verbal intelligence for different thresholds.*.*

| Variable |  | |  |  |  |  |  |  |  |  |  |
| --- | --- | --- | --- | --- | --- | --- | --- | --- | --- | --- | --- |
| wPLI | | | ImCOH | | | | | | | |  |
|  | *r* | p.value |  | | | *r* | | p.value | |  |  |
| APL_0.1 | .265 | .017 |  | APL_0.1 | | | .311 | | .007 | | |
| APL_0.2 | .262 | .019 |  | APL_0.2 | | | .316 | | .006 | | |
| APL_0.3 | .280 | .015 |  | APL_0.3 | | | .309 | | .007 | | |
| APL_0.4 | .285 | .017 |  | APL_0.4 | | | .31 | | .009 | | |
| APL_0.5 | .347 | .005 |  | APL_0.5 | | | .273 | | .035 | | |
| APL_0.6 | .363 | .017 |  | APL_0.6 | | | .3 | | .043 | | |
| CPL_0.1 | .272 | .014 |  | CPL_0.1 | | | .308 | | .007 | | |
| CPL_0.2 | .280 | .011 |  | CPL_0.2 | | | .31 | | .007 | | |
| CPL_0.3 | .285 | .010 |  | CPL_0.3 | | | .308 | | .007 | | |
| CPL_0.4 | .269 | .015 |  | CPL_0.4 | | | .304 | | .008 | | |
| CPL_0.5 | .261 | .019 |  | CPL_0.5 | | | .328 | | .004 | | |
| CPL_0.6 | .284 | .010 |  | CPL_0.6 | | | .311 | | .007 | | |
| D_0.1 | .291 | .008 |  | D_0.1 | | | .287 | | .013 | | |
| D_0.2 | .256 | .021 |  | D_0.2 | | | .307 | | .007 | | |
| D_0.3 | .226 | .043 |  | D_0.3 | | | .316 | | .006 | | |
| D_0.4 | .295 | .008 |  | D_0.4 | | | .33 | | .004 | | |
| D_0.5 | .265 | .017 |  | D_0.5 | | | .272 | | .018 | | |
| D_0.6 | .241 | .030 |  | D_0.6 | | | .302 | | .009 | | |
| Cl_0.1 | -0.255 | .022 |  | Cl_0.1 | | | .312 | | .006 | | |
| Cl_0.2 | -0.256 | .021 |  | Cl_0.2 | | | .315 | | .006 | | |
| Cl_0.3 | -0.254 | .022 |  | Cl_0.3 | | | .318 | | .005 | | |
| Cl_0.4 | -0.231 | .038 |  | Cl_0.4 | | | .308 | | .007 | | |
| Cl_0.5 | -0.285 | .010 |  | Cl_0.5 | | | .309 | | .007 | | |

*Note.*APL – Average Path Length . CPL – Characteristic Path Length. D – Diameter. Cl – Closeness Centrality. Only the estimates with p-value > 0.05 are displayed

Supplementary Figure 1. **The scatterplots for the relationship between the iMCOH-based sensor connectivity metrics and non-verbal intelligence**


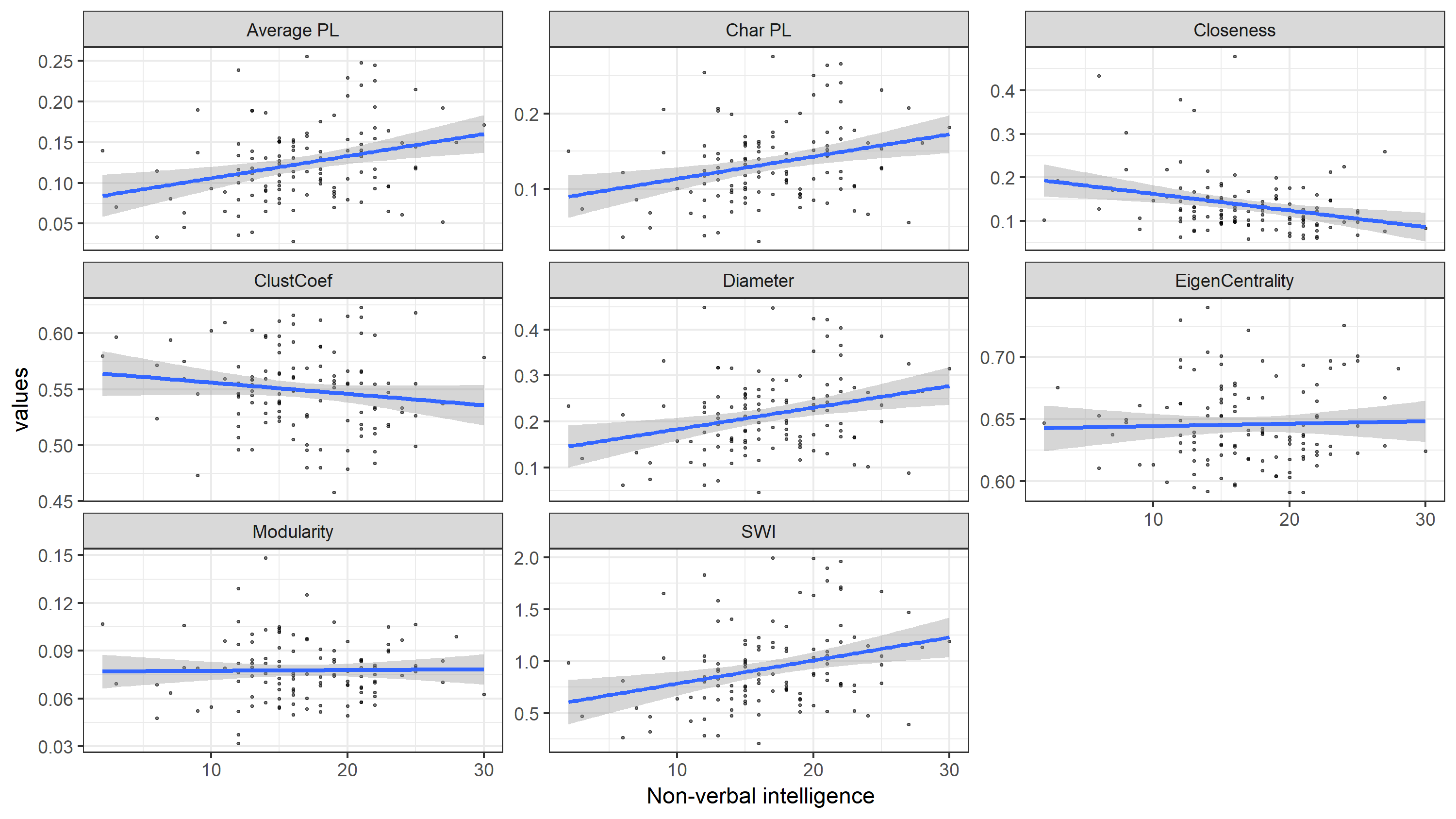


Supplementary Figure 2. **The scatterplots for the relationship between the iMCOH-based source connectivity metrics and non-verbal intelligence**


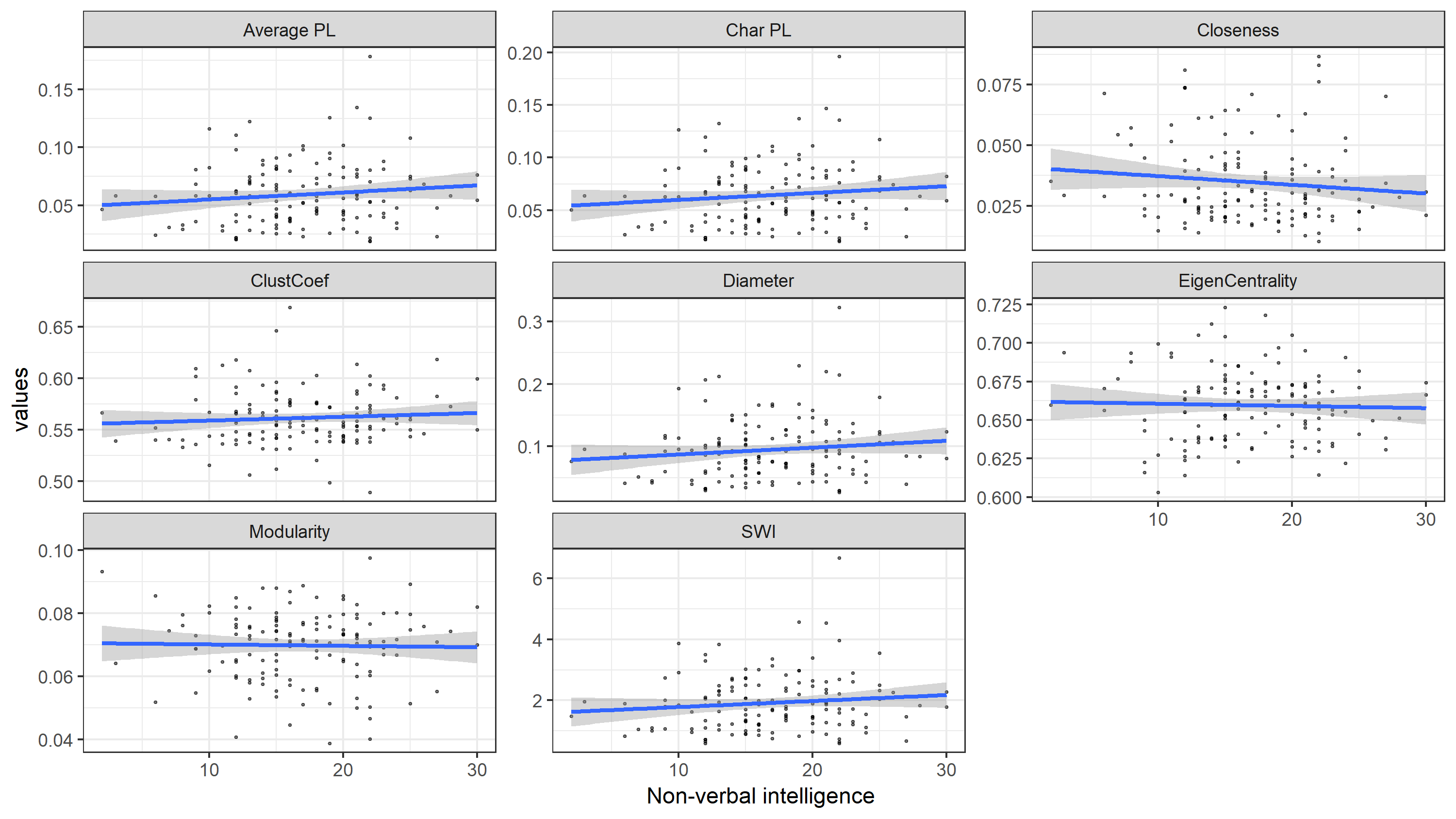

Supplement: Supplementary file 1 [file Table_1.docx]
